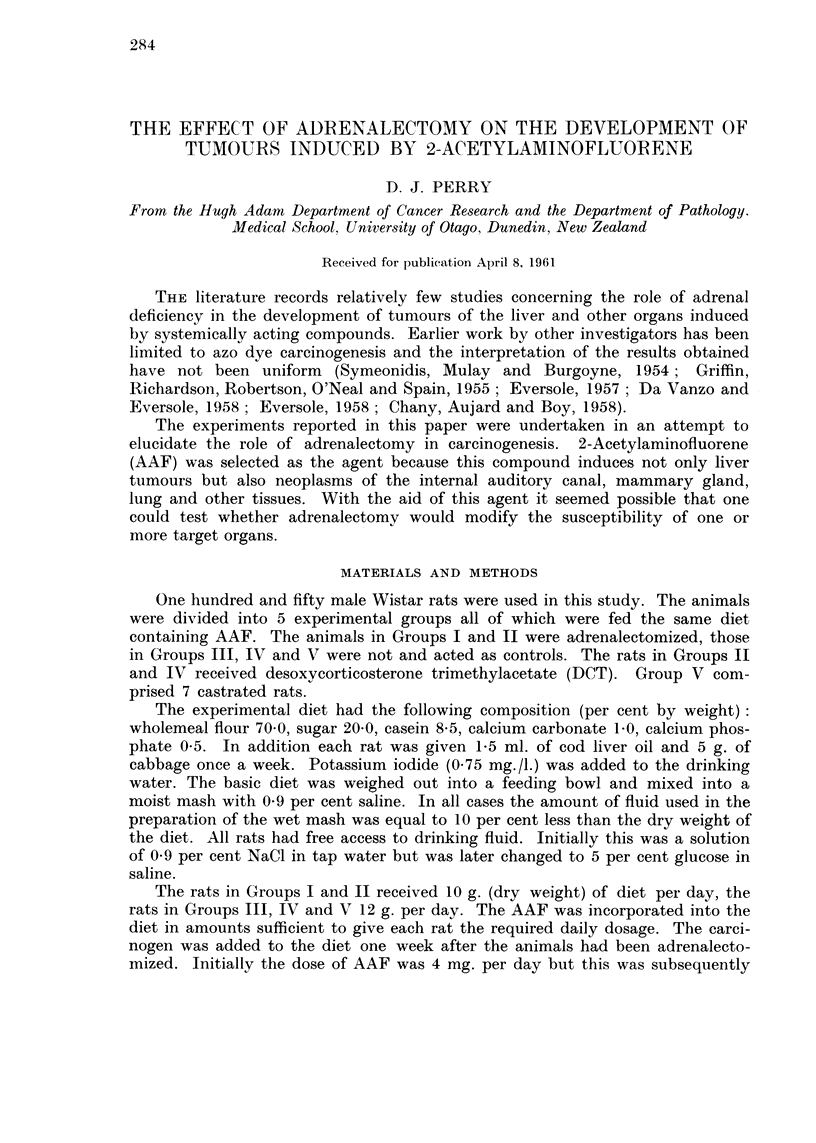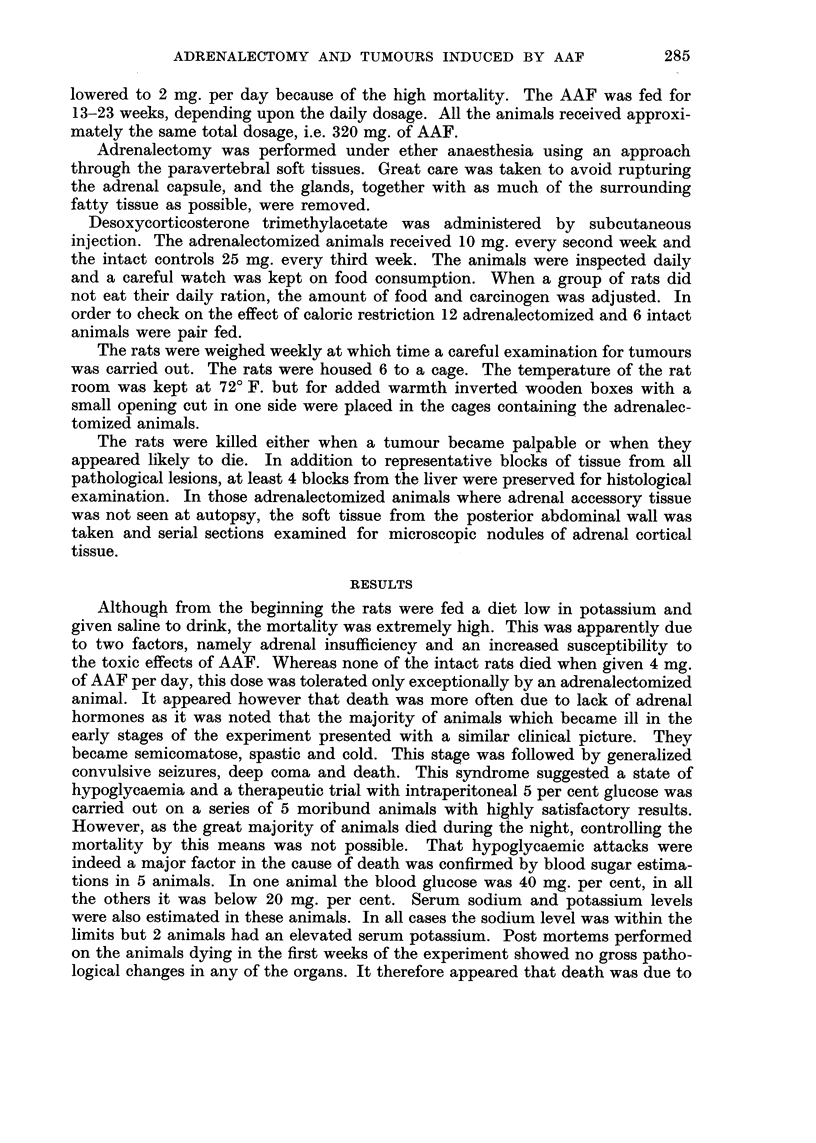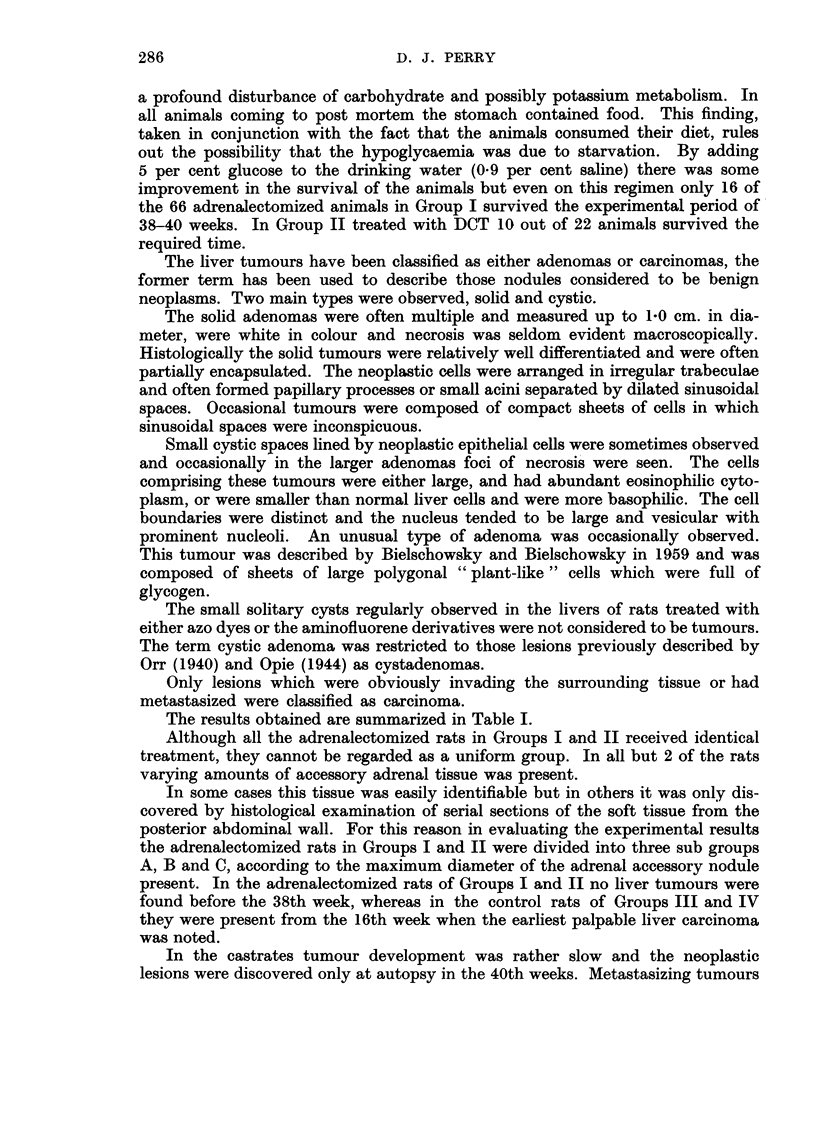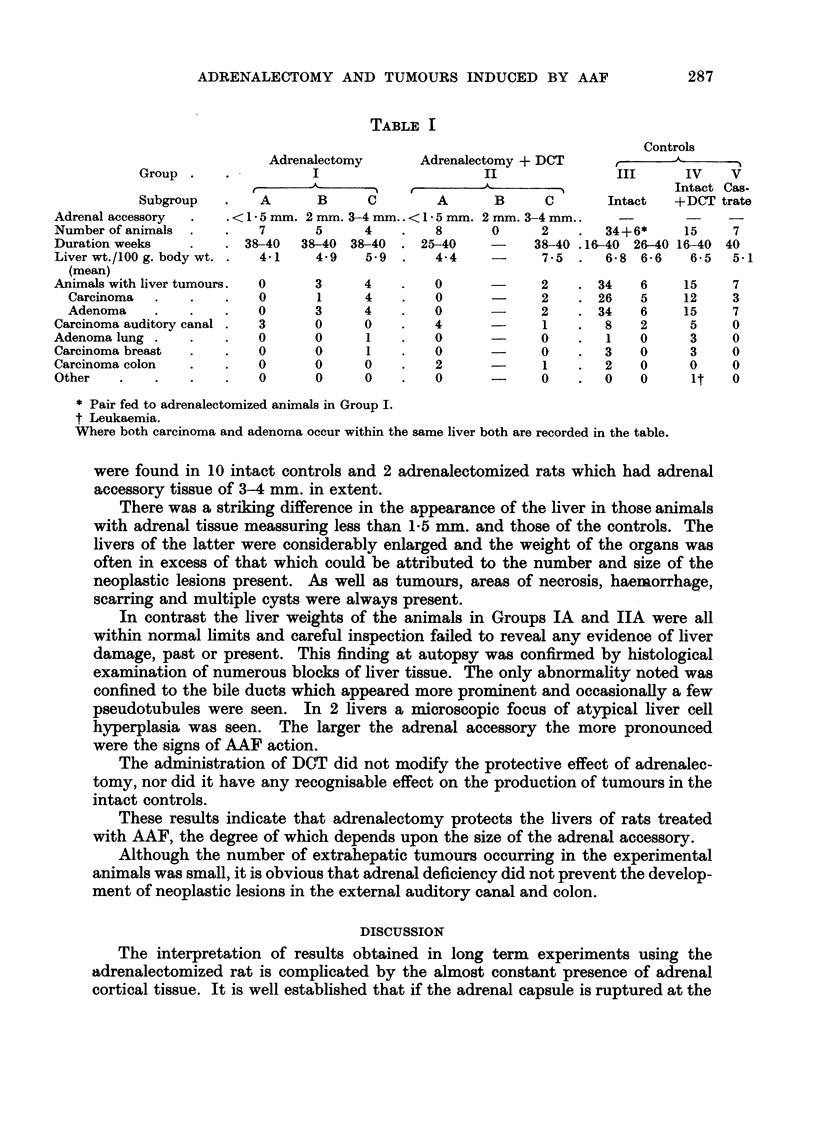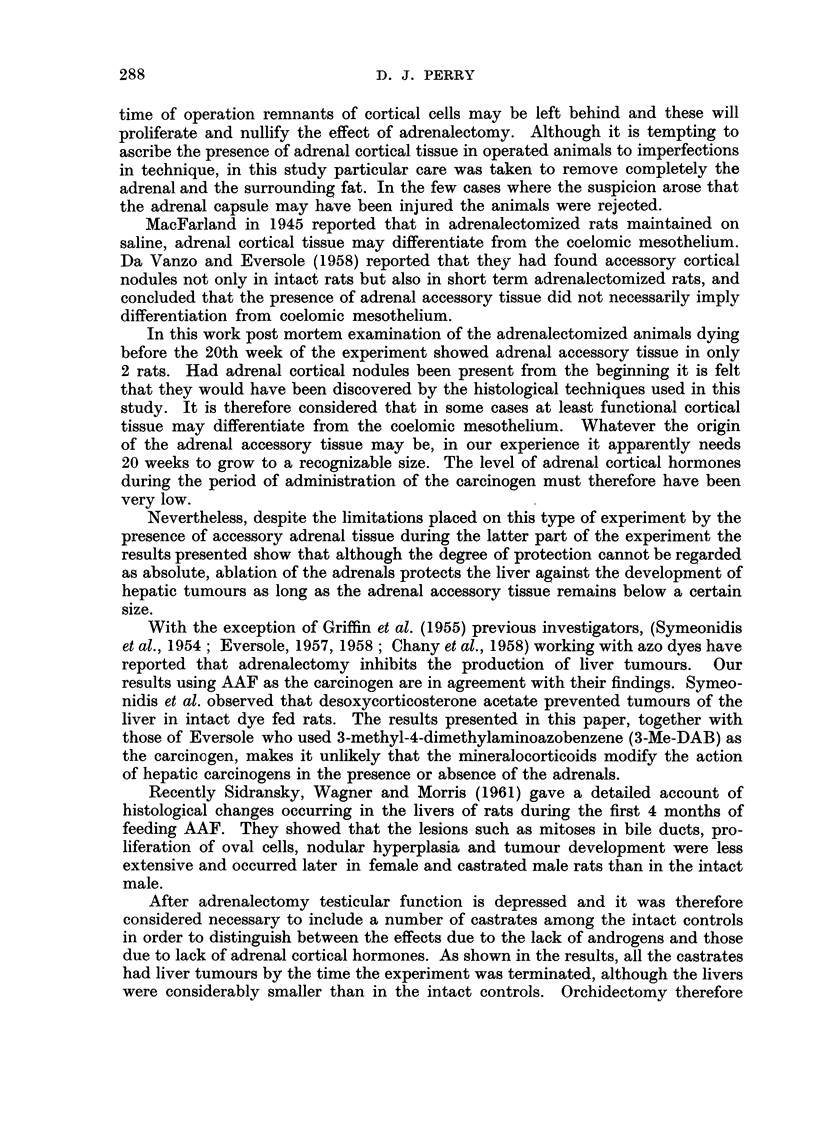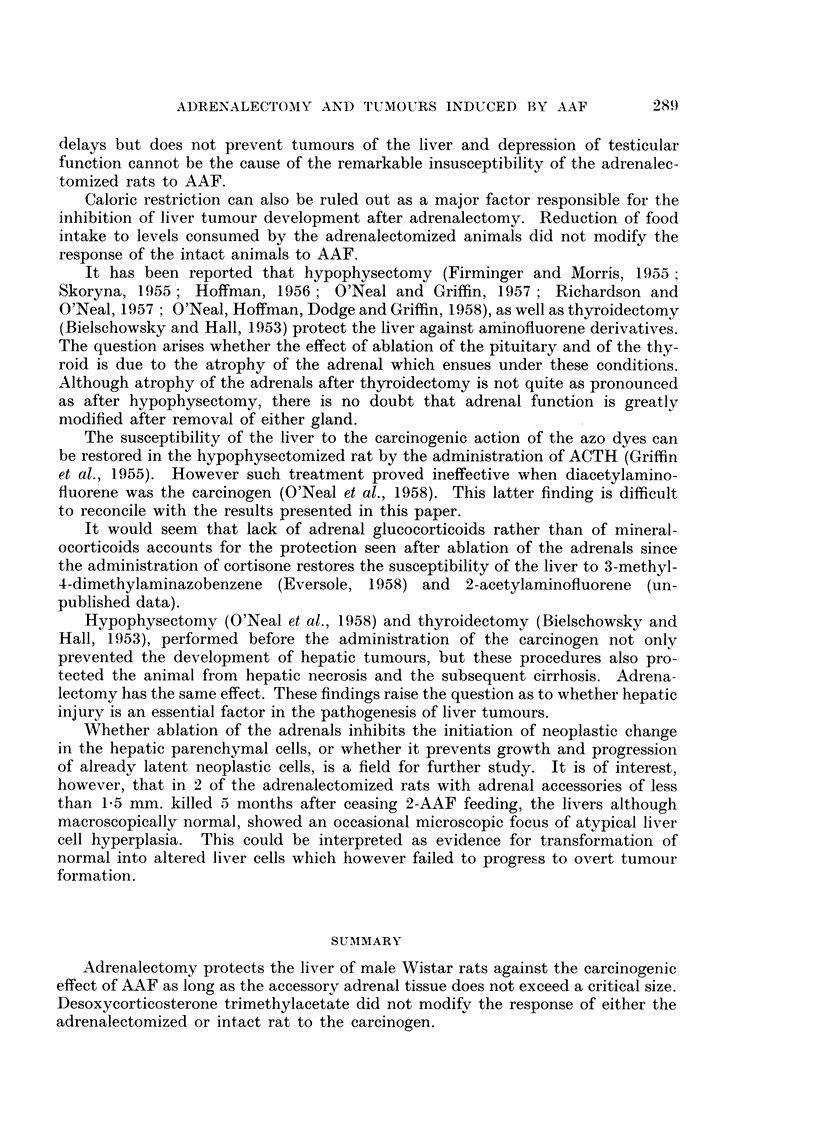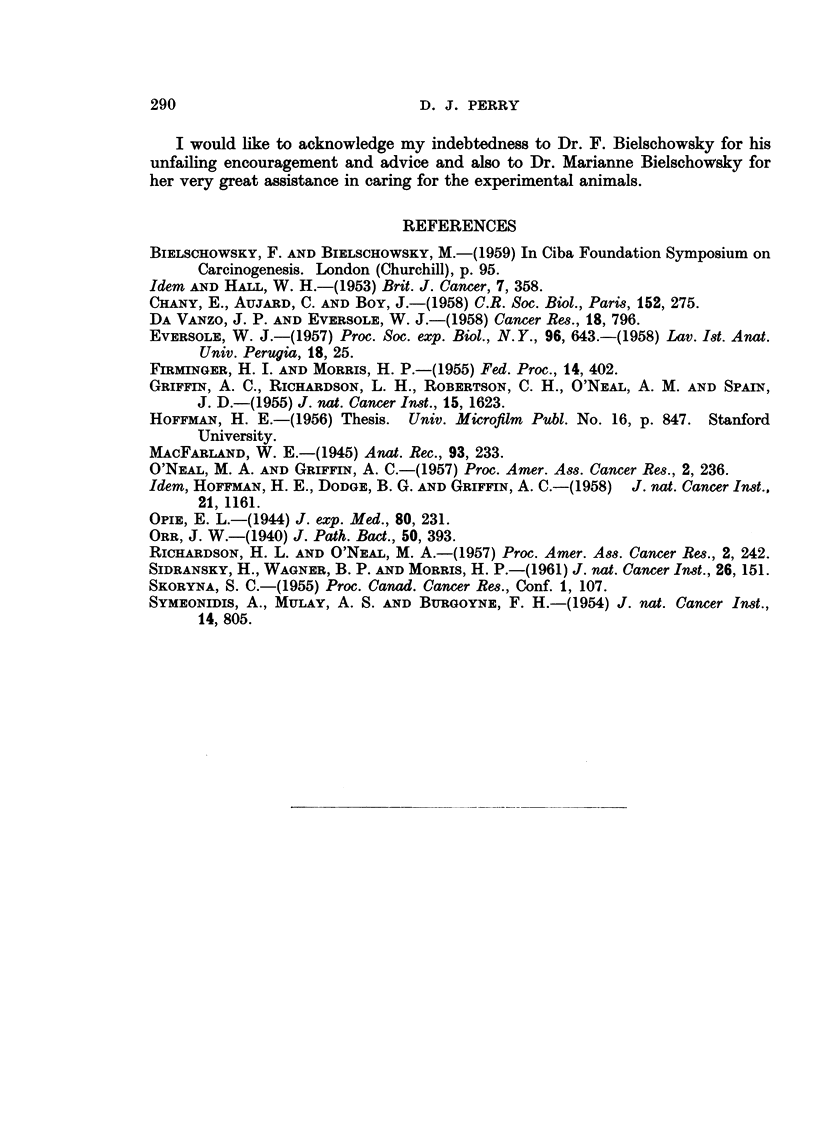# The Effect of Adrenalectomy on the Development of Tumours Induced by 2-acetylaminofluorene

**DOI:** 10.1038/bjc.1961.35

**Published:** 1961-06

**Authors:** D. J. Perry


### THE EFFECT OF ADRENALECTOMY ON THE DEVELOPMENT OF TUMOURS INDUCED BY 2-ACETYLAMINOFLUORENE

D. J. PERRY

*From the Hugh Adam Department of Cancer Research and the Department of Pathology, Medical School, University of Otago, Dunedin, New Zealand*

Received for publication April 8, 1961

THE literature records relatively few studies concerning the role of adrenal deficiency in the development of tumours of the liver and other organs induced by systemically acting compounds. Earlier work by other investigators has been limited to azo dye carcinogenesis and the interpretation of the results obtained have not been uniform (Symeonidis, Mulay and Burgoyne, 1954 ; Griffin, Richardson, Robertson, O'Neal and Spain, 1955 ; Eversole, 1957 ; Da Vanzo and Eversole, 1958 ; Eversole, 1958 ; Chany, Aujard and Boy, 1958).

The experiments reported in this paper were undertaken in an attempt to elucidate the role of adrenalectomy in carcinogenesis. 2-Acetylaminofluorene (AAF) was selected as the agent because this compound induces not only liver tumours but also neoplasms of the internal auditory canal, mammary gland, lung and other tissues. With the aid of this agent it seemed possible that one could test whether adrenalectomy would modify the susceptibility of one or more target organs.

### MATERIALS AND METHODS

One hundred and fifty male Wistar rats were used in this study. The animals were divided into 5 experimental groups all of which were fed the same diet containing AAF. The animals in Groups I and II were adrenalectomized, those in Groups III, IV and V were not and acted as controls. The rats in Groups II and IV received desoxycorticosterone trimethylacetate (DCT). Group V comprised 7 castrated rats.

The experimental diet had the following composition (per cent by weight) : wholemeal flour 70·0, sugar 20·0, casein 8·5, calcium carbonate 1·0, calcium phosphate 0·5. In addition each rat was given 1·5 ml. of cod liver oil and 5 g. of cabbage once a week. Potassium iodide (0·75 mg./l.) was added to the drinking water. The basic diet was weighed out into a feeding bowl and mixed into a moist mash with 0·9 per cent saline. In all cases the amount of fluid used in the preparation of the wet mash was equal to 10 per cent less than the dry weight of the diet. All rats had free access to drinking fluid. Initially this was a solution of 0·9 per cent NaCl in tap water but was later changed to 5 per cent glucose in saline.

The rats in Groups I and II received 10 g. (dry weight) of diet per day, the rats in Groups III, IV and V 12 g. per day. The AAF was incorporated into the diet in amounts sufficient to give each rat the required daily dosage. The carcinogen was added to the diet one week after the animals had been adrenalectomized. Initially the dose of AAF was 4 mg. per day but this was subsequently lowered to 2 mg. per day because of the high mortality. The AAF was fed for 13–23 weeks, depending upon the daily dosage. All the animals received approximately the same total dosage, i.e. 320 mg. of AAF.

Adrenalectomy was performed under ether anaesthesia using an approach through the paravertebral soft tissues. Great care was taken to avoid rupturing the adrenal capsule, and the glands, together with as much of the surrounding fatty tissue as possible, were removed.

Desoxycorticosterone trimethylacetate was administered by subcutaneous injection. The adrenalectomized animals received 10 mg. every second week and the intact controls 25 mg. every third week. The animals were inspected daily and a careful watch was kept on food consumption. When a group of rats did not eat their daily ration, the amount of food and carcinogen was adjusted. In order to check on the effect of caloric restriction 12 adrenalectomized and 6 intact animals were pair fed.

The rats were weighed weekly at which time a careful examination for tumours was carried out. The rats were housed 6 to a cage. The temperature of the rat room was kept at 72° F. but for added warmth inverted wooden boxes with a small opening cut in one side were placed in the cages containing the adrenalectomized animals.

The rats were killed either when a tumour became palpable or when they appeared likely to die. In addition to representative blocks of tissue from all pathological lesions, at least 4 blocks from the liver were preserved for histological examination. In those adrenalectomized animals where adrenal accessory tissue was not seen at autopsy, the soft tissue from the posterior abdominal wall was taken and serial sections examined for microscopic nodules of adrenal cortical tissue.

### RESULTS

Although from the beginning the rats were fed a diet low in potassium and given saline to drink, the mortality was extremely high. This was apparently due to two factors, namely adrenal insufficiency and an increased susceptibility to the toxic effects of AAF. Whereas none of the intact rats died when given 4 mg. of AAF per day, this dose was tolerated only exceptionally by an adrenalectomized animal. It appeared however that death was more often due to lack of adrenal hormones as it was noted that the majority of animals which became ill in the early stages of the experiment presented with a similar clinical picture. They became semicomatose, spastic and cold. This stage was followed by generalized convulsive seizures, deep coma and death. This syndrome suggested a state of hypoglycaemia and a therapeutic trial with intraperitoneal 5 per cent glucose was carried out on a series of 5 moribund animals with highly satisfactory results. However, as the great majority of animals died during the night, controlling the mortality by this means was not possible. That hypoglycaemic attacks were indeed a major factor in the cause of death was confirmed by blood sugar estimations in 5 animals. In one animal the blood glucose was 40 mg. per cent, in all the others it was below 20 mg. per cent. Serum sodium and potassium levels were also estimated in these animals. In all cases the sodium level was within the limits but 2 animals had an elevated serum potassium. Post mortems performed on the animals dying in the first weeks of the experiment showed no gross pathological changes in any of the organs. It therefore appeared that death was due to a profound disturbance of carbohydrate and possibly potassium metabolism. In all animals coming to post mortem the stomach contained food. This finding, taken in conjunction with the fact that the animals consumed their diet, rules out the possibility that the hypoglycaemia was due to starvation. By adding 5 per cent glucose to the drinking water (0·9 per cent saline) there was some improvement in the survival of the animals but even on this regimen only 16 of the 66 adrenalectomized animals in Group I survived the experimental period of 38–40 weeks. In Group II treated with DCT 10 out of 22 animals survived the required time.

The liver tumours have been classified as either adenomas or carcinomas, the former term has been used to describe those nodules considered to be benign neoplasms. Two main types were observed, solid and cystic.

The solid adenomas were often multiple and measured up to 1·0 cm. in diameter, were white in colour and necrosis was seldom evident macroscopically. Histologically the solid tumours were relatively well differentiated and were often partially encapsulated. The neoplastic cells were arranged in irregular trabeculae and often formed papillary processes or small acini separated by dilated sinusoidal spaces. Occasional tumours were composed of compact sheets of cells in which sinusoidal spaces were inconspicuous.

Small cystic spaces lined by neoplastic epithelial cells were sometimes observed and occasionally in the larger adenomas foci of necrosis were seen. The cells comprising these tumours were either large, and had abundant eosinophilic cytoplasm, or were smaller than normal liver cells and were more basophilic. The cell boundaries were distinct and the nucleus tended to be large and vesicular with prominent nucleoli. An unusual type of adenoma was occasionally observed. This tumour was described by Bielschowsky and Bielschowsky in 1959 and was composed of sheets of large polygonal "plant-like" cells which were full of glycogen.

The small solitary cysts regularly observed in the livers of rats treated with either azo dyes or the aminofluorene derivatives were not considered to be tumours. The term cystic adenoma was restricted to those lesions previously described by Orr (1940) and Opie (1944) as cystadenomas.

Only lesions which were obviously invading the surrounding tissue or had metastasized were classified as carcinoma.

The results obtained are summarized in Table I.

Although all the adrenalectomized rats in Groups I and II received identical treatment, they cannot be regarded as a uniform group. In all but 2 of the rats varying amounts of accessory adrenal tissue was present.

In some cases this tissue was easily identifiable but in others it was only discovered by histological examination of serial sections of the soft tissue from the posterior abdominal wall. For this reason in evaluating the experimental results the adrenalectomized rats in Groups I and II were divided into three sub groups A, B and C, according to the maximum diameter of the adrenal accessory nodule present. In the adrenalectomized rats of Groups I and II no liver tumours were found before the 38th week, whereas in the control rats of Groups III and IV they were present from the 16th week when the earliest palpable liver carcinoma was noted.

In the castrates tumour development was rather slow and the neoplastic lesions were discovered only at autopsy in the 40th weeks. Metastasizing tumours

TABLE I

| Group | Adrenalectomy I | | | Adrenalectomy + DCT II | | | Controls III | | Controls IV | Controls V |
|---|---|---|---|---|---|---|---|---|---|---|
| Subgroup | A | B | C | A | B | C | Intact | | Intact +DCT | Cas-trate |
| Adrenal accessory | <1·5 mm. | 2 mm. | 3–4 mm. | <1·5 mm. | 2 mm. | 3–4 mm. | — | | — | — |
| Number of animals | 7 | 5 | 4 | 8 | 0 | 2 | 34+6* | | 15 | 7 |
| Duration weeks | 38–40 | 38–40 | 38–40 | 25–40 | — | 38–40 | 16–40 | 26–40 | 16–40 | 40 |
| Liver wt./100 g. body wt. (mean) | 4·1 | 4·9 | 5·9 | 4·4 | — | 7·5 | 6·8 | 6·6 | 6·5 | 5·1 |
| Animals with liver tumours | 0 | 3 | 4 | 0 | — | 2 | 34 | 6 | 15 | 7 |
| Carcinoma | 0 | 1 | 4 | 0 | — | 2 | 26 | 5 | 12 | 3 |
| Adenoma | 0 | 3 | 4 | 0 | — | 2 | 34 | 6 | 15 | 7 |
| Carcinoma auditory canal | 3 | 0 | 0 | 4 | — | 1 | 8 | 2 | 5 | 0 |
| Adenoma lung | 0 | 0 | 1 | 0 | — | 0 | 1 | 0 | 3 | 0 |
| Carcinoma breast | 0 | 0 | 1 | 0 | — | 0 | 3 | 0 | 3 | 0 |
| Carcinoma colon | 0 | 0 | 0 | 2 | — | 1 | 2 | 0 | 0 | 0 |
| Other | 0 | 0 | 0 | 0 | — | 0 | 0 | 0 | 1† | 0 |

* Pair fed to adrenalectomized animals in Group I.
† Leukaemia.
Where both carcinoma and adenoma occur within the same liver both are recorded in the table.

were found in 10 intact controls and 2 adrenalectomized rats which had adrenal accessory tissue of 3–4 mm. in extent.

There was a striking difference in the appearance of the liver in those animals with adrenal tissue meassuring less than 1·5 mm. and those of the controls. The livers of the latter were considerably enlarged and the weight of the organs was often in excess of that which could be attributed to the number and size of the neoplastic lesions present. As well as tumours, areas of necrosis, haemorrhage, scarring and multiple cysts were always present.

In contrast the liver weights of the animals in Groups IA and IIA were all within normal limits and careful inspection failed to reveal any evidence of liver damage, past or present. This finding at autopsy was confirmed by histological examination of numerous blocks of liver tissue. The only abnormality noted was confined to the bile ducts which appeared more prominent and occasionally a few pseudotubules were seen. In 2 livers a microscopic focus of atypical liver cell hyperplasia was seen. The larger the adrenal accessory the more pronounced were the signs of AAF action.

The administration of DCT did not modify the protective effect of adrenalectomy, nor did it have any recognisable effect on the production of tumours in the intact controls.

These results indicate that adrenalectomy protects the livers of rats treated with AAF, the degree of which depends upon the size of the adrenal accessory.

Although the number of extrahepatic tumours occurring in the experimental animals was small, it is obvious that adrenal deficiency did not prevent the development of neoplastic lesions in the external auditory canal and colon.

### DISCUSSION

The interpretation of results obtained in long term experiments using the adrenalectomized rat is complicated by the almost constant presence of adrenal cortical tissue. It is well established that if the adrenal capsule is ruptured at the time of operation remnants of cortical cells may be left behind and these will proliferate and nullify the effect of adrenalectomy. Although it is tempting to ascribe the presence of adrenal cortical tissue in operated animals to imperfections in technique, in this study particular care was taken to remove completely the adrenal and the surrounding fat. In the few cases where the suspicion arose that the adrenal capsule may have been injured the animals were rejected.

MacFarland in 1945 reported that in adrenalectomized rats maintained on saline, adrenal cortical tissue may differentiate from the coelomic mesothelium. Da Vanzo and Eversole (1958) reported that they had found accessory cortical nodules not only in intact rats but also in short term adrenalectomized rats, and concluded that the presence of adrenal accessory tissue did not necessarily imply differentiation from coelomic mesothelium.

In this work post mortem examination of the adrenalectomized animals dying before the 20th week of the experiment showed adrenal accessory tissue in only 2 rats. Had adrenal cortical nodules been present from the beginning it is felt that they would have been discovered by the histological techniques used in this study. It is therefore considered that in some cases at least functional cortical tissue may differentiate from the coelomic mesothelium. Whatever the origin of the adrenal accessory tissue may be, in our experience it apparently needs 20 weeks to grow to a recognizable size. The level of adrenal cortical hormones during the period of administration of the carcinogen must therefore have been very low.

Nevertheless, despite the limitations placed on this type of experiment by the presence of accessory adrenal tissue during the latter part of the experiment the results presented show that although the degree of protection cannot be regarded as absolute, ablation of the adrenals protects the liver against the development of hepatic tumours as long as the adrenal accessory tissue remains below a certain size.

With the exception of Griffin *et al.* (1955) previous investigators, (Symeonidis *et al.*, 1954 ; Eversole, 1957, 1958 ; Chany *et al.*, 1958) working with azo dyes have reported that adrenalectomy inhibits the production of liver tumours. Our results using AAF as the carcinogen are in agreement with their findings. Symeonidis *et al.* observed that desoxycorticosterone acetate prevented tumours of the liver in intact dye fed rats. The results presented in this paper, together with those of Eversole who used 3-methyl-4-dimethylaminoazobenzene (3-Me-DAB) as the carcinogen, makes it unlikely that the mineralocorticoids modify the action of hepatic carcinogens in the presence or absence of the adrenals.

Recently Sidransky, Wagner and Morris (1961) gave a detailed account of histological changes occurring in the livers of rats during the first 4 months of feeding AAF. They showed that the lesions such as mitoses in bile ducts, proliferation of oval cells, nodular hyperplasia and tumour development were less extensive and occurred later in female and castrated male rats than in the intact male.

After adrenalectomy testicular function is depressed and it was therefore considered necessary to include a number of castrates among the intact controls in order to distinguish between the effects due to the lack of androgens and those due to lack of adrenal cortical hormones. As shown in the results, all the castrates had liver tumours by the time the experiment was terminated, although the livers were considerably smaller than in the intact controls. Orchidectomy therefore delays but does not prevent tumours of the liver and depression of testicular function cannot be the cause of the remarkable insusceptibility of the adrenalectomized rats to AAF.

Caloric restriction can also be ruled out as a major factor responsible for the inhibition of liver tumour development after adrenalectomy. Reduction of food intake to levels consumed by the adrenalectomized animals did not modify the response of the intact animals to AAF.

It has been reported that hypophysectomy (Firminger and Morris, 1955; Skoryna, 1955; Hoffman, 1956; O'Neal and Griffin, 1957; Richardson and O'Neal, 1957; O'Neal, Hoffman, Dodge and Griffin, 1958), as well as thyroidectomy (Bielschowsky and Hall, 1953) protect the liver against aminofluorene derivatives. The question arises whether the effect of ablation of the pituitary and of the thyroid is due to the atrophy of the adrenal which ensues under these conditions. Although atrophy of the adrenals after thyroidectomy is not quite as pronounced as after hypophysectomy, there is no doubt that adrenal function is greatly modified after removal of either gland.

The susceptibility of the liver to the carcinogenic action of the azo dyes can be restored in the hypophysectomized rat by the administration of ACTH (Griffin *et al.*, 1955). However such treatment proved ineffective when diacetylaminofluorene was the carcinogen (O'Neal *et al.*, 1958). This latter finding is difficult to reconcile with the results presented in this paper.

It would seem that lack of adrenal glucocorticoids rather than of mineralocorticoids accounts for the protection seen after ablation of the adrenals since the administration of cortisone restores the susceptibility of the liver to 3-methyl-4-dimethylaminazobenzene (Eversole, 1958) and 2-acetylaminofluorene (unpublished data).

Hypophysectomy (O'Neal *et al.*, 1958) and thyroidectomy (Bielschowsky and Hall, 1953), performed before the administration of the carcinogen not only prevented the development of hepatic tumours, but these procedures also protected the animal from hepatic necrosis and the subsequent cirrhosis. Adrenalectomy has the same effect. These findings raise the question as to whether hepatic injury is an essential factor in the pathogenesis of liver tumours.

Whether ablation of the adrenals inhibits the initiation of neoplastic change in the hepatic parenchymal cells, or whether it prevents growth and progression of already latent neoplastic cells, is a field for further study. It is of interest, however, that in 2 of the adrenalectomized rats with adrenal accessories of less than 1·5 mm. killed 5 months after ceasing 2-AAF feeding, the livers although macroscopically normal, showed an occasional microscopic focus of atypical liver cell hyperplasia. This could be interpreted as evidence for transformation of normal into altered liver cells which however failed to progress to overt tumour formation.

### SUMMARY

Adrenalectomy protects the liver of male Wistar rats against the carcinogenic effect of AAF as long as the accessory adrenal tissue does not exceed a critical size. Desoxycorticosterone trimethylacetate did not modify the response of either the adrenalectomized or intact rat to the carcinogen.

I would like to acknowledge my indebtedness to Dr. F. Bielschowsky for his unfailing encouragement and advice and also to Dr. Marianne Bielschowsky for her very great assistance in caring for the experimental animals.

### REFERENCES

Bielschowsky, F. and Bielschowsky, M.—(1959) In Ciba Foundation Symposium on Carcinogenesis. London (Churchill), p. 95.

*Idem* and Hall, W. H.—(1953) *Brit. J. Cancer*, **7**, 358.

Chany, E., Aujard, C. and Boy, J.—(1958) *C.R. Soc. Biol., Paris*, **152**, 275.

Da Vanzo, J. P. and Eversole, W. J.—(1958) *Cancer Res.*, **18**, 796.

Eversole, W. J.—(1957) *Proc. Soc. exp. Biol., N.Y.*, **96**, 643.—(1958) *Lav. Ist. Anat. Univ. Perugia*, **18**, 25.

Firminger, H. I. and Morris, H. P.—(1955) *Fed. Proc.*, **14**, 402.

Griffin, A. C., Richardson, L. H., Robertson, C. H., O'Neal, A. M. and Spain, J. D.—(1955) *J. nat. Cancer Inst.*, **15**, 1623.

Hoffman, H. E.—(1956) Thesis. *Univ. Microfilm Publ.* No. 16, p. 847. Stanford University.

MacFarland, W. E.—(1945) *Anat. Rec.*, **93**, 233.

O'Neal, M. A. and Griffin, A. C.—(1957) *Proc. Amer. Ass. Cancer Res.*, **2**, 236.

*Idem*, Hoffman, H. E., Dodge, B. G. and Griffin, A. C.—(1958) *J. nat. Cancer Inst.*, **21**, 1161.

Opie, E. L.—(1944) *J. exp. Med.*, **80**, 231.

Orr, J. W.—(1940) *J. Path. Bact.*, **50**, 393.

Richardson, H. L. and O'Neal, M. A.—(1957) *Proc. Amer. Ass. Cancer Res.*, **2**, 242.

Sidransky, H., Wagner, B. P. and Morris, H. P.—(1961) *J. nat. Cancer Inst.*, **26**, 151.

Skoryna, S. C.—(1955) *Proc. Canad. Cancer Res.*, Conf. **1**, 107.

Symeonidis, A., Mulay, A. S. and Burgoyne, F. H.—(1954) *J. nat. Cancer Inst.*, **14**, 805.